# Interferon-Independent Upregulation of Interferon-Stimulated Genes during Human Cytomegalovirus Infection is Dependent on IRF3 Expression

**DOI:** 10.3390/v11030246

**Published:** 2019-03-12

**Authors:** Caroline L. Ashley, Allison Abendroth, Brian P. McSharry, Barry Slobedman

**Affiliations:** Department of Infectious Diseases and Immunology, Charles Perkins Centre, University of Sydney, Camperdown, New South Wales 2050, Australia; cash7683@uni.sydney.edu.au (C.L.A.); allison.abendroth@sydney.edu.au (A.A.); brian.mcsharry@sydney.edu.au (B.P.M.)

**Keywords:** interferon, human cytomegalovirus, IRF3, ISG15, interferon stimulated genes

## Abstract

The antiviral activity of type I interferons (IFNs) is primarily mediated by interferon-stimulated genes (ISGs). Induction of ISG transcription is achieved when type I IFNs bind to their cognate receptor and activate the Janus Kinase/Signal Transducer and Activator of Transcription (JAK/STAT) signaling pathways. Recently it has become clear that a number of viruses are capable of directly upregulating a subset of ISGs in the absence of type I IFN production. Using cells engineered to block either the response to, or production of type I IFN, the regulation of IFN-independent ISGs was examined in the context of human cytomegalovirus (HCMV) infection. Several ISGs, including IFIT1, IFIT2, IFIT3, Mx1, Mx2, CXCL10 and ISG15 were found to be upregulated transcriptionally following HCMV infection independently of type I IFN-initiated JAK-STAT signaling, but dependent on intact IRF3 signaling. ISG15 protein regulation mirrored that of its transcript with IFNβ neutralization failing to completely inhibit ISG15 expression post HCMV infection. In addition, no detectable ISG15 protein expression was observed following HCMV infection in IRF3 knockdown CRISPR/Cas-9 clones indicating that IFN-independent control of ISG expression during HCMV infection of human fibroblasts is absolutely dependent on IRF3 expression.

## 1. Introduction

Human cytomegalovirus (HCMV) is a betaherpesvirus with a 40–90% seroprevalence worldwide [1,2,3,4,5,6]. Infection poses a serious risk to those that are immunocompromised or immunonaïve [4,5,7,8,9,10,11,12]. Type I interferons (IFNα and IFNβ) play a crucial role in the innate immune response to HCMV, with viral replication inhibited following pre-treatment with type I IFN [13,14,15] and significantly enhanced in cells with an abrogated type I IFN response [16].

Initiation of the type I IFN response to HCMV is multifaceted. It begins when the viral envelope glycoproteins (gB, gL and gH) that initiate virion attachment are detected by Toll-like receptor 2 (TLR-2) [17,18]. TLR-2 stimulation activates a signaling cascade that culminates in initiation of type I IFN transcription, dependent on activation of a number of key transcription factors including interferon regulatory factor 3 (IRF3) [19,20], IRF7 [21] and NFκB [22]. This IFN production pathway is stimulated again when HCMV enters the cell; dsDNA viral genomes released into the cytoplasm are detected by sensors such as cGAS [23], IFI16 [24] and ZBP1 [25]. Each of these sensors is capable of activating the Stimulator of IFN Genes (STING) leading to phosphorylation of IRF3 and type I IFN production [23,26,27].

The cognate receptor of type I IFNs is the cell surface IFN alpha/beta receptor (IFNAR). When type I IFNs bind to the IFNAR, transcription of their associated ISGs are stimulated through the Janus Kinase/Signal Transducer and Activator of Transcription (JAK/STAT) signaling pathway [28] resulting in the induction of an antiviral state [29,30]. Although this is the canonical mechanism of ISG upregulation several reports have indicated that HCMV infection can regulate ISG expression in the absence of *de novo* protein synthesis i.e., without IFN production. Studies of HCMV infection in the presence of cycloheximide (CHX) have shown upregulation of IFIT1 (IFI56), IFIT2 (ISG54), IFIT3 (cig49, ISG60), MxA (the protein produced from the Mx1 gene) and ISG15 [31,32,33]. These are some of the best studied ISGs and, during HCMV infection, expression of transcripts for many of these genes are also unaffected by CHX treatment [20,34] suggesting that HCMV infection can drive ISG transcription in an IFN-independent manner. Depletion of IRF3 levels using specific siRNAs before infection with HCMV in the presence of CHX resulted in a marked decrease in ISG production compared with a non-specific control siRNA, indicating that this transcription factor can play an important role in HCMV-mediated ISG regulation that occurs independently of de novo protein synthesis [20]. It is becoming increasingly clear in multiple other virus infections that a number of well-known ISGs can be upregulated directly by infection without the requirement for IFN production [35,36,37]. Therefore, we initiated a study examining the expression of key ISGs that are potentially IFN-independent during HCMV infection to more precisely define their mechanism(s) of regulation.

## 2. Materials and Methods

### 2.1. Cell Culture, Viral Infection and Treatment of Cells with Conditioned Supernatants

HEK293T cells (ATCC), HFF-1 primary human foreskin fibroblasts (HFs) (sourced from ATCC), human telomerase-immortalized fibroblasts (hTERT HFs) [38] and HFs engineered to express the nPro protein of bovine viral diarrhea virus (nPro/HFs) or the V protein of parainfluenza virus 5 (V/HFs) as previously described [16,39] were grown at 37 °C and 5% CO_2_ in DMEM media supplemented with 10% foetal calf serum (FCS) and penicillin streptomycin (100 units/mL). The low passage clinical isolate Merlin (used in all HCMV infections in this study) was generated from a bacterial artificial chromosome (BAC) pAL1111 as described previously [40]. Virus stocks were generated from the supernatant of infected HFs. Supernatant was collected when all HFs in infected flasks displayed cytopathic effect (CPE). Supernatants were spun at 845× g for 10 min to pellet cell debris before a second centrifugation at 21875× g in an ultracentrifuge for 2 h to pellet the virus (Thermo Scientific™ A-621 6 Fixed-Angle Rotor, Thermo Scientific™ Sorvall™ WX+ ultracentrifuge). Concentrated virus pellets were resuspended in fresh supplemented DMEM and stored at −80 °C.

Ultraviolet (UV) irradiation of virus was performed by applying 720 mJ/cm^2^ of UV using a CL-1000 Ultraviolet Crosslinker (UVP, now Analytik Jena). To confirm successful inactivation of virus, UV irradiated virus was added to fresh monolayers of HFs and development of CPE was not observed. Cell-free viable or UV-irradiated virus was applied to cultures for 90 minutes before being washed off, this point was taken as time zero of the infection.

For treatment with supernatants from infected parental cells, supernatants were harvested at 24 h post infection (h.p.i.) and stored at −80°C. Prior to use supernatants were filtered (0.1 μm) to remove any contaminating infectious virions (approximately 230 nm in diameter [41]). Supernatants were diluted 1:1 with cell culture media before being applied to uninfected cells. Supernatant IFNβ quantification was performed by enzyme-linked immunosorbent assay (ELISA) (elisakit.com, Melbourne, Australia).

IFNβ neutralization was achieved by pre-treating cells and the treatments to be applied to them (virus, infected cell supernatant or recombinant IFNβ) for 1 h with 100 neutralisation units of anti-IFNβ rabbit polyclonal Ab (AB1431, Merck Millipore, Sydney, Australia). Polyclonal rabbit IgG was used as a control (Sigma-Aldrich, Sydney, Australia).

### 2.2. Quantitative Reverse Transcription Polymerase Chain Reaction (qRT-PCR)

Total RNA was extracted from HFs using an innuPREP RNA minikit (Analytik-Jena, Jena, Germany) prior to cDNA synthesis using the AffinityScript cDNA synthesis kit (Aligent Technologies, Santa Clara, CA, USA). For polymerase chain reaction (PCR), reaction mixtures were created using Brilliant II SYBR Green qPCR Master Mix (Agilent Technologies) and relative levels of mRNA expression recorded by qRT-PCR (Roche LightCycler® 480 Instrument II PCR machine) at 50 °C for 2 min followed by 10 min at 95 °C for denaturation then 50 amplification cycles of 15 s at 95 °C and 45 s at 60°C, finally melt curve data was generated through 1 min at 95 °C, 30 s at 50 °C, 30 s at 95 °C. Test gene mRNA levels were normalized to mRNA levels of the housekeeping gene glyceraldehyde-3-phosphate dehydrogenase (*GAPDH*). Specific primers used in this study were the following: *GAPDH*-F, TCACCAGGGCTGCTTTTAAC; *GAPDH*-R 5′- ACAAGCTTCCCGTTCTCAG -3′; *ISG15*-F 5′-GCGAACTCATCTTTGCCAGTA-3′; *ISG15*-R 5′-AGCATCTTCACCGTCAGGTC-3′; *Gal-9*-F 5′-CTTTCATCACCACCATTCTG-3′; *Gal-9*-R 5′-ATGTGGAACCTCTGAGCACTG-3′; *viperin*-F 5′-AGCAGCTGGTCCTGAGAGG-3′; *viperin*-R 5′-TGGCTCTCCACCTGAAAAGT-3′; *IFIT1*-F 5′-GCCTAATTTACAGCAACCATGA-3′; *IFIT1*-R 5′-TCATCAATGGATAACTCCCATGT-3′; *IFIT2*-F 5′-ACGTCAGCTGAAGGGAAACA-3′, *IFIT2*-R 5′-TTAGTTGCCGTAGGCTGCTC-3′, *IFIT3*-F 5′- AGAGACACAGAGGGCAGTCA-3′; *IFIT3*-R 5′-GGCATTTCAGCTGTGGAAGG-3′; *Mx1*-F 5′-CTCCGACACGAGTTCCACAA-3′, *Mx1*-R 5′-GGCTCTTCCAGTGCCTTGAT-3′; *Mx2*-F 5′-TGATTTCTCCATCCTGAACGTG-3′; *Mx2*-R 5′-GGGCCTTAGACATGTGCTGT-3′; *CXCL10*-F 5′- GAAAGCAGTTAGCAAGGAAAGGT-3′; *CXCL10*-R 5′-GACATATACTCCATGTAGGGAAGTGA-3′.

### 2.3. Western Blot

Protein lysates were extracted from cells, mixed with SDS loading buffer and boiled for 5 min before Western blot analysis. Protein expression was detected with anti-*ISG15* (1:1000, #2743, Cell Signaling, Beverly, MA, USA), anti-IRF3 (1:1000, sc-376455, Santa Cruz Biotechnology, Dallas, TX, USA) and anti-*GAPDH* (FL-335) (1:2000, sc-25778, Santa Cruz, Biotechnology, Dallas, TX, USA). The secondary antibody used on all blots was horseradish peroxidase-lined Donkey anti-rabbit IgG (1:2000, 406401, Biolegend, San Diego, CA, USA). All blots were developed with Clarity™ Western ECL Substrate (Bio-Rad, Hercules, CA, USA) before imaging with a Bio-Rad ChemiDoc™ MP.

### 2.4. CRISPR/Cas-9

Genome editing using CRISPR/Cas-9 was performed with the dual-vector lentivirus GeCKO system as described previously [42]. Briefly, Cas-9 expressing lentivirus was harvested from the supernatant (filtered, 0.45 μm pore size) of HEK293T cells transfected with the packaging plasmid pCMV8.91, expression plasmid lentiCas-9-Blast and envelope plasmid pMD2G; 50% confluent telomerase immortalized (hTERT HFs), chosen for their longevity, were transduced with this lentivirus in the presence of 5 μg/mL polybrene. Successfully transduced hTERTs were selected with 5μg/mL Blasticidin. Next these Cas-9 hTERT HFs were transduced with a lentivirus expressing guide RNA (gRNA) specific for the desired target gene, in this case IRF3. To generate a gRNA specific for IRF3 the following pair of DNA oligomers were annealed and ligated into the lentiguide-Puro expression plasmid following Esp3I (BsmBI) digestion: 5′- CACCG**GAGGTGACAGCCTTCTACCG** -3′ and 5′- AAAC**CGGTAGAAGGCTGTCACCTCC**-3′. The resulting gRNA (in bold) was designed to target a protein-coding region of IRF3 [43]. This expression plasmid was transfected into HEK293T cells with the same packaging and envelope plasmids as the Cas-9 expressing lentivirus (pCMV8.91 and pMD2G, respectively). Lentivirus was extracted from the filtered (0.45 μm pore size) supernatant of these cells and applied to 50% confluent Cas-9 hTERT HFs in the presence of 5 μg/mL polybrene. Cells successfully transduced with the gRNA lentivirus were then selected for with 1 μg/mL puromycin. In order to create clones, selected cells were seeded into 96 well plates at the approximate concentration of 0.5 cells/well, minimizing the chance of two cells ending up in the same well. Plates were monitored for growth over a 3-week period. Individual clones were identified and expanded before testing for IRF3 expression by immunoblot.

## 3. Results

### 3.1. Transcript Upregulation of Interferon (IFN)-Independent Interferon-Stimulated Genes (ISGs) Following Infection with Human Cytomegalovirus (HCMV) and Ultraviolet-Irradiated HCMV (UV-HCMV) in IRF3-Deficient Human Foreskin Fibroblasts (HFs)

**Figure 1:**
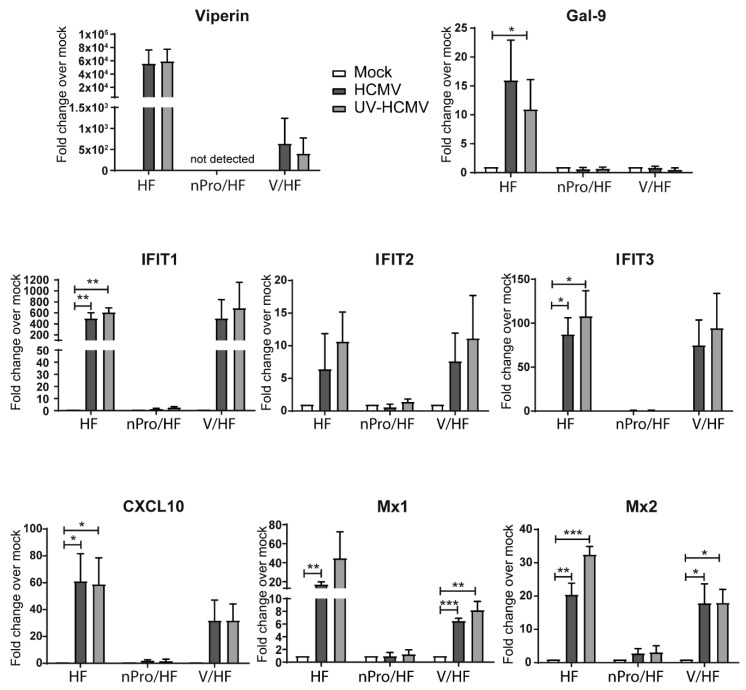
Regulation of interferon-stimulated gene (ISG) transcript levels following infection with intact or ultraviolet (UV)-irradiated human cytomegalovirus (HCMV) in cells with an abrogated interferon (IFN) response. Primary human foreskin fibroblasts (HFs), nPro expressing HFs (nPro/HFs) and V protein-expressing HFs (V/HFs) were infected in parallel with HCMV or UV-HCMV at an MOI of 3. 6 h.p.i. RNA was extracted, converted to cDNA and the relative levels of various ISG transcripts (normalised to the housekeeping gene *GAPDH*) were calculated. Individual bars represent the average fold change in transcript level compared to the mock infection for each cell type (set to 1). Error bars indicate the SEM and statistical significance was determined using a Student’s two-tailed *t*-test, *n* = 3, * *p* < 0.05, ** *p* < 0.01, *** *p* < 0.001, **** *p* < 0.0001.

**Figure 2:**
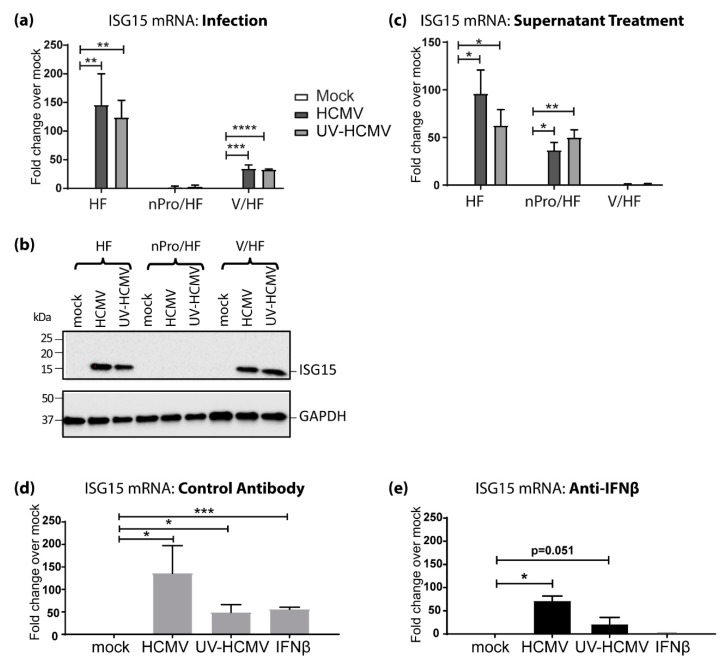
IRF3-dependent, STAT1-independent regulation of ISG15 following HCMV infection (**a**) Primary HFs, nPro/HFs and V/HFs were infected at an MOI of 3 with HCMV or UV-HCMV and levels of ISG15 transcript were analyzed 6 h.p.i as in Figure 1. (**b**) Primary HFs, nPro/HFs and V/HFs were infected at an MOI of 3 with HCMV or UV-HCMV. Protein lysates were harvested at 24 h.p.i. and analyzed by immunoblot, staining for ISG15 and GAPDH. (**c**) Primary HFs, nPro/HFs and V/HFs were treated with filtered (0.1 μm pore size) supernatant from mock, HCMV or UV-HCMV infected primary HFs. RNA harvested at 6 h post-treatment was analyzed by quantitative reverse transcription polymerase chain reaction (qRT-PCR) as in Figure 1. RNA was extracted at 6 h.p.i. from primary HFs treated with 100 U of IFNβ or infected at a multiplicity of infection (MOI) of 3 with HCMV or UV-HCMV in the presence of (**d**) a control antibody or (**e**) IFNβ-neutralizing antibody and analyzed by qRT-PCR. Error bars indicate the standard error of the mean (SEM) and statistical significance was calculated using a Student’s two-tailed T-test. *n* = 3, * *p* < 0.05, ** *p* < 0.01, *** *p* < 0.001, **** *p* < 0.0001.

To investigate IFN-independent ISG regulation during HCMV infection we utilized previously generated cell lines [16,39] based on the characterized abilities of the nPro protein of bovine viral diarrhea virus (BVDV) to target IRF3 (blocking IFNβ production) [44] and of the V protein of parainfluenza virus type 5 (PIV-5) to target STAT1 (blocking IFN responsiveness) [45]. RNA was harvested from mock, HCMV or ultraviolet-irradiated HCMV (UV-HCMV) infected primary HFs, nPro/HFs or V/HFs at 6 h post infection (h.p.i.). The levels of specific ISG transcripts were then quantified by qRT-PCR (Figure 1). Transcript levels of *viperin* were highly elevated (≈ 6 × 10^4^ fold) in the primary HFs following infection with both intact and UV-irradiated HCMV. In V/HFs *viperin* transcript levels were also increased with both intact and UV-HCMV but to a lesser extent (≈ 5 × 10^2^ fold). This finding demonstrates that, whilst the STAT-1-dependent signaling that initiates ISG transcription following IFNAR binding plays a significant role in *viperin* induction by HCMV, an IFN-independent pathway is also involved. In the nPro expressing HFs, that lack functional IRF3 expression, *viperin* transcript was undetectable. *Viperin* is an ISG known to be upregulated by HCMV in an IRF3-dependent, IFN-independent manner [20,46,47,48,49] and so this pattern of regulation was expected. On the other hand, the induction of *Gal-9* transcription by HCMV and UV-HCMV which is known to be dependent on both IRF3-mediated type I IFN production and JAK/STAT signaling in this system [39], was elevated post infection in the primary HFs but not in either of the IFN-abrogated cell lines (Figure 1). We have previously observed this pattern of regulation for IFN-dependent ISGs *PML* and *Sp100* [50] in the same system. We extended our analysis to a range of ISGs that have previously been identified as being regulated directly by virus infection in an IFN-independent manner i.e., *IFIT1, IFIT2, IFIT3, ISG15, CXCL10, Mx1* and *Mx2* [20,33,34,35,36,37]. The regulation profile of all of the potentially IRF3-dependent, IFN-independent genes identified in our literature screen, i.e., *IFIT1, IFIT2, IFIT3, CXCL10, Mx1, Mx2* (Figure 1) and *ISG15* (Figure 2a) was similar to that observed for *viperin*, in that upregulation was observed in the primary HFs and V/HFs but not in the nPro/HFs. It is interesting to note that the extent of transcript upregulation in the V/HFs compared to the primary HFs varied from gene to gene, suggesting that some transcripts (*IFIT1, IFIT2, IFIT3*) were upregulated to the same extent in the presence or absence of interferon signaling whilst for others (*CXCL10, MX1, Mx2* and *ISG15*) both IFN-independent and dependent mechanisms contribute to maximal upregulation during infection. Taken together, these data demonstrate that upregulation of this subset of ISGs by HCMV infection can occur independently of viral gene expression, in a manner that is dependent on IRF3 but not dependent on STAT1.

### 3.2. IFN-Independent, IRF3-Dependent Induction of ISG15 Expression Contributes Significantly to Its Upregulation by HCMV Infection

Quantification of *ISG15* mRNA by qRT-PCR revealed significant IRF3-dependent, IFN signaling-independent upregulation during HCMV infection (Figure 2a). Therefore, we sought to investigate whether this regulation also occurred at the protein level. *ISG15* protein was undetectable in mock infected HFs, mock infected nPro/HFs and mock infected V/HFs. Infection with either HCMV or UV-HCMV potently upregulated *ISG15* in the primary HFs and in V/HFs but not in the nPro/HFs (Figure 2b). This mirrored the qRT-PCR results (Figure 2a) and reinforced the importance of IRF3 in *ISG15* regulation by HCMV. Supernatant taken from HCMV-infected primary HFs was able to upregulate *ISG15* transcript in primary HFs and nPro/HFs but not in V/HFs (Figure 2c). This demonstrates firstly, that nPro/HFs are capable of upregulating *ISG15* transcript and secondly that there is a soluble factor produced during HCMV infection that can induce *ISG15* upregulation in a STAT1-dependent, IRF3-independent manner, most likely type I IFN. In primary HFs, exposure to recombinant IFNβ resulted in a significant upregulation of *ISG15* mRNA similar to that caused by UV-HCMV (Figure 2d). While the increase in *ISG15* transcription driven by IFNβ could be blocked by an IFNβ neutralizing antibody, the increase in *ISG15* mRNA caused by either HCMV or UV-HCMV was not (Figure 2e). Taken together these data demonstrate that whilst soluble IFNβ is a potent driver of STAT-1-dependent *ISG15* upregulation during HCMV infection, there is a significant component of *ISG15* expression mediated by a type-I IFN signaling-independent, IRF3-dependent mechanism.

### 3.3. IRF3 Knockout (KO) by CRISPR/Cas-9 Inhibits ISG15 Protein Expression during HCMV Infection Mirroring the Phenotype Seen in nPro/HFs

Due to the fact that the nPro/HFs rely on the expression of an exogenous viral protein to target IRF3 expression, it is conceivable that there may be effects other than targeting IRF3 for proteasomal degradation [44] that could contribute to regulation of IFN-independent ISGs. In this respect, nPro is also known to impair expression of a number of cellular proteins key to the IFN response including *TRIF, TBK1, Mda-5* and *RIG-I* [44]. For this reason we performed additional experiments whereby we specifically targeted IRF3 expression using the CRISPR/Cas-9 system.

**Figure 3:**
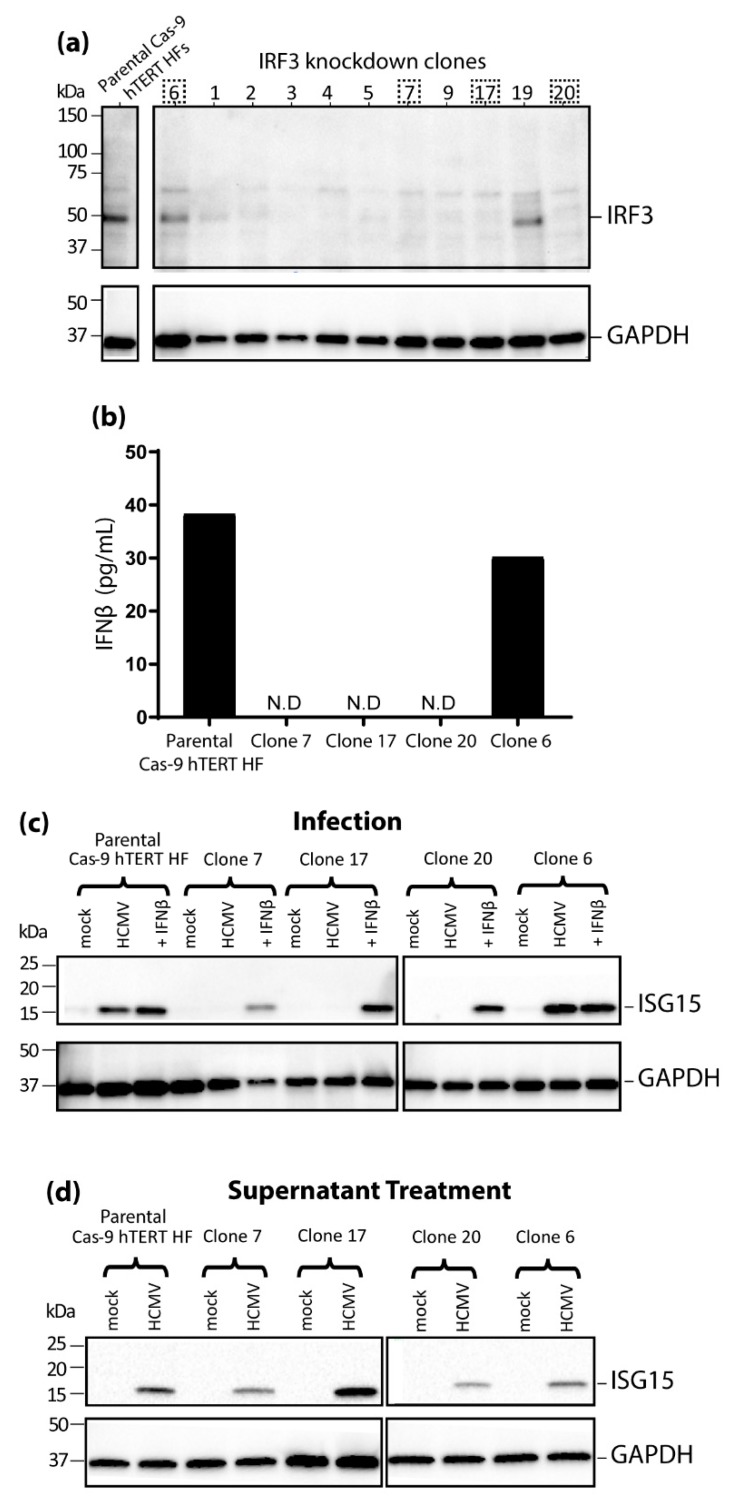
IFN-independent, IRF3-dependent regulation of ISG15 recapitulated in IRF3 knockout (KO) CRISPR/Cas-9 engineered telomerase immortalized (hTERT) HFs. (**a**) IRF3 KO clones generated by CRISPR/Cas-9 were screened by immunoblot for IRF3 alongside the parental Cas-9 hTERT HFs with GAPDH as a loading control. The dashed boxes denote which clones were chosen for further experiments. (**b**) Supernatants were collected from the Cas-9 hTERT HFs and individual clones after 24 h of HCMV infection (MOI 3) before IFNβ was quantified by an enzyme-linked immunosorbent assay (ELISA) (N.D: not detected indicates IFNβ levels less than the lower limit of quantification 5 pg/mL). (**c**) Successful IRF3 KO clones 7, 17 and 20 were infected with HCMV (MOI of 3) or treated with recombinant IFNβ (100U) in parallel with unsuccessful clone 6, and the parental Cas-9 hTERT HFs. Protein lysates were extracted at 24 h.p.i. before immunoblotting for *ISG15* and *GAPDH*. (**d**) Individual clones and parental Cas-9 hTERT HFs were treated with supernatant from HCMV infected cells for 24 h before immunoblotting for *ISG15* and *GAPDH*.

Using the dual-vector lentivirus GeCKO system [42] we transduced telomerase-immortalized hTERT HFs [38] first with a Cas-9 expressing lentivirus and then with a lentivirus designed to express a guide RNA specific for IRF3. Clones of the transduced hTERT HFs were then isolated and screened for IRF3 expression. Immunoblotting for IRF3 indicated that the majority of the clones derived from the CRISPR/Cas-9 targeting of IRF3 successfully knocked down IRF3 protein expression (Figure 3a), and we selected three of these (clone 7, 17 and 20) for subsequent experiments. There were also two clones (6 and 19) in which IRF3 knockdown was unsuccessful (Figure 3a). As these unsuccessful clones had been through the same lentiviral transduction and clone expansion process as those which had yielded knocked down IRF3 expression, we chose one of these (clone 6) to be included alongside the parental Cas-9 hTERT HFs as an additional negative control in subsequent experiments.

When IFNβ production was measured by ELISA 24 h.p.i with HCMV, the parental cells and clone 6 produced 38.3 pg/mL and 30.2 pg/mL, respectively, whilst the levels in the successful IRF3 knockdown clones (7, 17 and 20) were below the lower limit of quantitation of the assay (Figure 3b) consistent with functional disruption of IRF3 expression. In accordance with this, at 24 h.p.i. there was no detectable *ISG15* protein expression in the successful IRF3 knockdown clones (7, 17 and 20) whilst *ISG15* could be readily observed in the parental Cas-9 hTERT HFs and in the unsuccessful clone 6 (Figure 3c) infected with HCMV. However, treatment with either IFNβ alone (Figure 3c) or supernatant isolated from infected parental Cas-9 hTERT HFs (Figure 3d) was able to induce robust *ISG15* protein expression in all cells tested indicating that cells lacking IRF3 expression retain the capacity to upregulate *ISG15*. Together these results directly demonstrate the essential role IRF3 plays in HCMV-induced *ISG15* upregulation in both an IFN-dependent and IFN-independent manner.

## 4. Discussion

These data presented here demonstrate that IRF3-dependent, IFN-independent upregulation of *IFIT1, IFIT2, IFIT3, CXCL10, Mx1, Mx2* and *ISG15* can occur during HCMV infection. This may explain why CHX fails to inhibit expression of these genes during HCMV infection [31,32,33,34] and corroborates data demonstrating significant inhibition of transcription of each of these genes by IRF3-specific siRNA [20]. Induction of the IFN-independent ISG viperin during HCMV infection is known to be induced by either IRF3 or IRF1, binding directly to its promoter [20,32,46,48], and this may also be the mechanism of *IFIT1, IFIT2, IFIT3, CXCL10, Mx1, Mx2* and *ISG15* upregulation. Indeed, microarray analysis has identified *IFIT2, IFIT3*, and *ISG15* and *viperin* as likely IFN-independent ISGs [50] and the same study demonstrated that *IFIT1* transcript upregulation can be induced directly by expression of constitutively active *IRF3* [51]. Additionally, IRF3 binding sites are present in the promoter regions of *IFIT1, IFIT2, IFIT3, Mx2* and *ISG15* (as determined by the Transcription Factor analysis tool within the Monash University Interferome database [51]).

In our study, the requirement of IRF3 for HCMV-induced *ISG15* upregulation was confirmed in IRF3 knockout CRISPR Cas-9 hTERT HFs. In primary HFs significant upregulation of *ISG15* transcript was observed following HCMV infection even in the presence of IFNβ blocking antibodies. These two observations combine to definitively demonstrate that the upregulation of *ISG15* caused by HCMV infection can occur in an IFN-independent, IRF3-dependent manner. This finding is an essential addition to the study of *ISG15* in the context of not only HCMV infection but other viruses as well. It adds complexity to recent work describing *ISG15* as an antiviral effector during HCMV infection that is antagonized by IE1, pUL50, pUL26 and pUL25 [27,52,53,54], and potentially explains the persistence of *ISG15* even in cells treated with high concentrations of the JAK inhibitor pyridone-6 [27].

IFN-independent induction of *IFIT1, IFIT2, IFIT3, CXCL10, Mx1, Mx2* and *ISG15* is not limited to HCMV and may also be IRF3-dependent in response to other viruses. Certainly, this appears to be the case for *IFIT1/ISG56* whose expression can be induced following treatment with UV-irradiated herpes simplex virus (HSV) 1 in murine cells in the absence of IRF9 but not IRF3 [55]. There is a strong case for IRF3-dependent, IFN-independent upregulation of several other ISGs investigated in this study with other viruses too: *IFIT2* upregulation has been observed following HSV-1 infection in cell lines with mutations in the JAK/STAT signaling pathway [31]; human immunodeficiency virus (HIV) can induce *Mx1/MxA* expression independently of IFN [56]; and *IFIT1* upregulation has been observed in response to vesicular stomatitis virus (VSV), encephalomyocarditis virus, or Sendai virus infection in cells incapable of responding to type I IFNs (JAK1-defecient p2.1 cells) [37]. Direct ISG upregulation by IRF3 activation could be an essential contributor to the intrinsic, IFN-independent host response to these viruses.

In a broader sense, the results of our study raise questions about the role of IFN-independent ISGs in host responses to HCMV. *ISG15* appears to be primarily antiviral [27,52,53,54] however, *viperin* is known to enhance viral replication by allowing HCMV to regulate cellular lipid metabolism [32,57]. This is not the case for other viruses as *viperin* mainly functions as an antiviral ISG [58,59], and demonstrates the potential for investigations into IFN-independent ISGs to illustrate new functions of well-known host defense proteins.

The fact that IFN-independent ISG regulation occurs suggests that it is likely to benefit the virus and/or the host. Perhaps, if the ISG in question has been co-opted to benefit the virus (as is the case with viperin) the lack of requirement for IFN means that the virus can manipulate the host to accommodate replication early in infection. Whilst if the ISGs in question are antiviral, IFN-independent ISG induction could be a mechanism used to induce an antiviral response in an environment where an influx of proinflammatory IFN could be detrimental. To this end it would be relevant to investigate whether the IFN-independent ISG regulation caused by HCMV infection is cell type-dependent as it appears to be for *IFIT2* following infection with HSV-1 [33]. It is also possible that in non-fibroblastic cell types, IFN-independent ISG induction may depend on transcription factors other than IRF3 e.g. IRF1 or NF-κB may be involved. In the case of viperin, IRF1 is also known to induce transcription directly by binding to its promoter [48].

Finally, it should be noted that a number of the IFN-independent ISGs examined in this paper are among the best studied ISGs and are often used as hallmarks of IFN production. Perhaps this is because their direct, IFN-independent upregulation following infection, coupled with their IFN-dependent induction, means they are present at higher levels following infection than those that are only induced in an IFN-dependent manner. Based on the data here and in previous reports, care should be exercised in using production of such ISGs as a hallmark of IFN production in the context of viral infection.
